# Reproductive Health Right Practice among Preparatory School Female Students of Assela Town, Arsi Zone, Oromia Regional State, Ethiopia

**DOI:** 10.1155/2020/6070638

**Published:** 2020-09-28

**Authors:** Mesfin Tafa Segni, Tigist Tafa, Hailu Fekadu, Shimelis Adugna, Meselech Assegid

**Affiliations:** ^1^Department of Public Health, College of Health Sciences, Arsi University, Assela, Ethiopia; ^2^Schools of Public Health, Addis Ababa University, Addis Ababa, Ethiopia

## Abstract

**Introduction:**

Knowledge and practice toward reproductive health right (RHR) is critical to protect young women, especially school girls, from unwanted reproductive outcomes as improving access to reproductive health services. However, the majority of young people including female secondary school students in Ethiopia have very little knowledge on the youth's reproductive health rights. The aim of this study was to assess knowledge and practice toward reproductive health right among preparatory female students in Assela Town, Arsi Zone, Ethiopia.

**Methods:**

A study was conducted among 403 preparatory school female students in Assela Town. Simple random sampling was employed to select the subjects, and a self-administered questionnaire was used to collect data. The collected data were entered using EPI Info version 3.5.4 and exported to SPSS version 21 for analysis. Descriptive and logistic regression analysis was carried out.

**Result:**

Sixty percent of girls discussed reproductive issues openly with their peers. About 94% of the respondents knew, at least, one contraceptive method; injectable (91.2%) was the most known type of contraceptives. Marital status, father occupation, discussion on sexual and reproductive issues, and having sexual partners were affecting the practice of reproductive health rights.

**Conclusions:**

Knowledge of the students was moderate on reproductive health right which was 70%. Practice of sexual and reproductive health rights was 22.6% among the study participants. It is recommended that promotion on sexual and reproductive health right through media is important.

## 1. Introduction

Reproductive health right is the right of couples or every person to decide freely and responsibly on their own reproductive conditions and sexuality. The 1994 International Conference on Population Development in Cairo (ICPD) gave more emphasis on improving gender equality, empowering women, decreasing violence; and controling fertility [1, 2]. The 4th World Conference in Beijing in 1995 set a declaration and platform for action aimed at equality and opportunity for women. Some of the strategic objective were as follows: revise laws and policies to ensure women's equal right and access to economic resources, ensure equal access to education, eradicate women illiteracy, improve participation and decision making, and decrease all forms of discrimination [3].

From a total of 1.5 billion youths and adolescents globally, 78% live in the low socioeconomic continents such as Asia and Africa [4]. Sexual and reproductive health (SRH) problems, particularly unsafe sex, is the second leading cause of disease, disability, or death in developing countries where effective interventions are available to solve the problem [5]. Knowledge of the youth about reproductive health and the right of access to reproductive health right is critical to their ability to protect themselves from unwanted reproductive outcomes [6].

Globally, 7% to 48% adolescent girls and 0.2% to 32% of adolescent boys reported that their first sexual intercourse was forced [7]. Increasing violations against the sexual and reproductive right is confirmed by the frequent incidences that have been highlighted by the media, and few of which have reached the court [8]. Sexual abuses reported from 19 countries range from 7 to 34% for girls and 3 to 29% for boys. Every five minutes, a young person commits suicide, often due to problems related to sexual and RH, such as sexual violence [9].

In Africa, reproductive health right is hidden and unrecognized. Adolescents are the neglected group of population to reproductive health services. Poor organization of youth-friendly services at health facilities or youth clubs, lack of availability and affordability of reproductive health commodity, long waiting time, and confidentiality are the main factors that affect getting the reproductive health services. Violence against women and adolescents, especially on girls, is the big problem of the world. A study conducted in Nigeria showed that, among high-school students who had knowledge of contraception, only 5% used any contraceptive methods [6].

Many young adolescents in sub-Saharan Africa do not know how to protect themselves and their partners against HIV/AIDS and other sexually transmitted infections (STI). Thus, more than half of all new HIV infection and other STIs occur in people between the age of 15 and 24 years. HIV/AIDS is now the leading cause of death in women aged 15–29 years [10, 11]. In Ethiopia, the level of knowledge of students about reproductive and sexual rights was found to be low, where there is a high fertility rate and it is almost from the women under the age of 25 years. Half of all births are among under-fifteen, and more than one in three births are among 15–19 years [12].

Studies conducted in university and preparatory students in Ethiopia showed that 63.7% of students say that a married woman has no right to limit the number of children without her husband's consent and 63.7% of students agreed that the parents have the right to decide on sexual and reproductive issues of their children [13]. Childbearing begins early in Ethiopia; more than 34% of women gave birth by the age of 18, and more than half (54%), by the age of 20 (EDHS 2011). Another study showed that 45% of the total births in the country occur among adolescent girls and young women and 60% of adolescent pregnancies are unwanted or unintended [14]. In most developing countries such as Ethiopia, sexual and reproductive health right is not well studied, especially among unmarried girls. Therefore, this study was aimed to assess reproductive health right knowledge and practice of reproductive health and associated factors among female students in Assela Town preparatory schools, Arsi Zone, Ethiopia.

## 2. Methodology

### 2.1. Study Setting

A cross-sectional study was conducted among female students of Assela preparatory schools, Assela Town, Arsi Zone, Oromia Regional State, Ethiopia, in 2015. Assela Town is located 175 km to the south east of Addis Ababa (capital city of Ethiopia). There were two governmental preparatory schools in the town. The total number of students in the two schools registered as regular for the year 2015 was 2617 (females = 1087 (41.5%)).

#### 2.1.1. Study Population

Female students aged 18–24 years who were registered for the year 2015.

#### 2.1.2. Exclusion Criteria

Sick students during data collection, students learning at night, and students of age <18 and >24 years.

### 2.2. Sample Size and the Sampling Procedure

The sample size was calculated based on a single population proportion using the prevalence of knowledge of reproductive health right from a study concucted in Wollaita Sodo University which was 54.4% [15]. Adding 5% nonresponse rate, the sample was 403.

There are only two governmental preparatory schools in the town; they were selected on the basis of availability. The sample was allocated proportionally among the schools.

In order to select samples, firstly, the list of female students was prepared from the registered list of each selected class, and the participants were selected through the systematic random sampling method.

### 2.3. Data Collection Tools and Quality Assurance

The English version of the questionnaire was developed based on the relevant literature on the subject. Then, it was translated to local language Amharic and Afan Oromo for simplicity, and both were used for data collection. Data were collected using the self-administered questionnaire. The selected students for the study were informed about the objective of the study and called to the auditorium of each school, and then, questionnaires were distributed for the study participants. Finally, facilitators collected the filled questionnaires and checked for completeness and consistency of the questionnaires. Training was given for facilitators for a day on the overall procedure of data collection. A pretest was conducted on 50 students, and some modification was performed.

### 2.4. Operational Definitions

✓ *Knowledge of RHR*. The knowledge of students on reproductive health information, specifically on unwanted pregnancy, contraceptive, violence, STIs, the decision of marriage, and discussion on reproductive health issues. Students who responded for the given question below the mean score were considered as having poor knowledge, whereas those who scored above the mean were considered as having good knowledge.
✓ *Practice of RHR*. In this study, practice refers to the utilization of any type contraceptive methods.

### 2.5. Data Analysis

The collected data were rechecked for completeness before data entry. Three hundred and ninety-three completed questionnaires were coded and entered into EPI Info version 3.5.1 statistical software and exported to SPSS version 21 for performing satirical analysis. Ten incomplete questionnaires were discarded. Descriptive statistics such as frequency and percentages were used. Logistic regression was applied to identify the associated factor with knowledge of reproductive health right and practice (utilization of contraceptives). Bivariate analysis was used to calculate the crude odds ratio (COR). Then, multivariate analysis was run to see the independent effect of each variable, and the Adjusted Odds ratio (AOR) was calculated; an arbitrary *p* value of <0.30 was used as the criterion to include variables in the multivariable logistic regression model to control confounding effects, and the results were considered statically significant at a *p* value 0.05. Independent variables were recoded when deemed during analysis of logistic regression.

### 2.6. Ethical Considerations

Ethical clearance was obtained from the Research and Ethics Committee (REC) of the School of Public Health, Addis Ababa University. Furthermore, cooperation was obtained from the zonal educational office of Assela Town and the director of the schools. Verbal informed consent was obtained after explaining the purpose of the study, and they had full right to decide to participate or not or to skip questions they do not want to respond. Confidentiality of the information was maintained by excluding names as identification on the questionnaire and keeping their privacy during the data collection.

## 3. Result

### 3.1. Sociodemographic Characteristics of the Respondents

Out of the 403 respondents, 393 respondents completed the given questionnaire with a response rate of 97.5%. The mean ± (SD) age of the respondents was 18.3 ± 0.57 years. About 70% of the respondents were Orthodox Christians. Two hundred and thirty-eight respondents were urban dwellers, and 43.3% were living together with their parents. Concerning the educational status of their parents, the majority of them attended primary education. Around 35% of their fathers were government workers, and 149 (37.9%) of them were farmers, while more than half (51.1%) of their mothers were housewives. The sociodemographic characteristics of the participants are displayed in Table 1.

### 3.2. Knowledge of Reproductive Health Rights

Seventy percent of respondents were knowledgeable about reproductive health rights. Media such as TV/radio were the most reported source of information. Absence of discussion with parents (51.7%) and clubs (28.4%) in schools were the two predominantly mentioned reasons for the deficiency of information on reproductive health issues. With regard to the problem of unsafe sexual practice, unwanted pregnancy 340 (92.1%) and STI 339 (91.9%) were the most reported problems by the respondents.

The majority (84.7%) of the respondents agree with the youth's right of their own mate selection without their family approval. In this study, 321 (81.7%) of the respondents agree on confidentiality of reproductive health services. More than two-thirds of the respondents disagree on the idea of a married woman's right to refuse to have children if she does not want. Two hundred and eighty-two (71.8%) of the respondents agree on the refusal of a woman to have sex regardless of her husband's wishes (Table 2).

### 3.3. Knowledge About Sexually Transmitted Infection (STI) and Contraceptives

With regard to the respondents' knowledge about STI, 97.2% of them know, at least, one type of STI, where HIV (382 (97.2%)) was the most known STI followed by gonorrhoea (314 (82.2%)). The majority of (94.9%) of the respondents know ways of preventing unwanted pregnancy, and using contraceptives (307 (87.7%)) followed by abstinence (283 (80.9%)) were the most mentioned methods of prevention. Ninety-four participants mentioned, at least, one type of contraceptive methods where injectable was the most known (340 (91.2%)) method, followed by pills (334 (89.5%)) (Table 3).

### 3.4. Sexual and Reproductive Health *t* Practice

Over half (54.7%) of the respondents had no sexual partner at the time of the survey, but 52.9% of them have never had a sexual experience.

Fifty-five percent of the girls had discussions on sexual and reproductive issues, and of these, 60% had discussions with peers. Few respondents (22.6%) used modern contraceptives, whereas pills (24 (27.8%)) were the most utilized contraceptive followed by injectable (21 (23.6%)). Regarding the source of contraceptives, clinics (33 (36.3%)) and pharmacy (26 (28.6%)) were the most used sources of the contraceptive methods for the girls, while shops (5 (5.5%)) were the least used source of contraceptives for the respondents (Table 4).

### 3.5. Associated Factors with Knowledge on Reproductive Health Rights

Bivariate analysis was run to see the association of sociodemographic characteristics and knowledge on reproductive health right; however, none of them showed a significant association. In the bivariate analysis, knowing safe time from pregnancy and knowledge of ways of prevention method of unwanted pregnancy, knowledge on problem of unsafe sex, information about contraceptives, discussion on sexual and reproductive issues, and having sexual partners were significantly associated with knowledge on reproductive health rights. In the multivariate analysis only, knowing safe time of pregnancy and preventing unwanted pregnancy remained significant. Those girls who knew safe time of pregnancy were 1.64 times more likely to know reproductive health rights than who did not know (AOR = 1.636; 95% CI = 1.006, 2.659). Knowing ways of preventing unwanted pregnancy increases the odds of knowledge on reproductive health rights by more than 3 times (AOR = 3.277; 95% CI = 1.461, 7.351), as depicted in Table 5.

### 3.6. Associated Factors with Practice on Reproductive Health Rights

Contraceptive use was taken as an outcome variable from reproductive health right practice. In the bivariate analysis; marital status, place of residence, living with, father's occupation and education, discussion on sexual and reproductive issues, and having sexual partners were significantly associated with contraceptive utilization. Respondents from the rural areas were 1.7 times more likely to utilize contraceptive than urban residents (COR = 1.700; 95% CI = 1.056, 2.738). Participants who were living with friends and alone were 3 times more likely to utilize modern contraceptives than those who were living with families.

In multivariate analysis, marital status, father's occupation, discussion on sexual and reproductive issues, and having sexual partners were characteristics that remained significant. Singles were 85% less likely to utilize modern contraceptives than the married (AOR = 0.155; 95% CI = 0.032, 0.762). Respondents from farming families were more than 6 times more likely to utilize contraceptives (AOR = 6.137; 95% CI = 1.945, 19.369) than the employed. Respondents who did not discuss on the sexual and reproductive matter were 60% less likely to practice reproductive health rights than those who discuss (AOR = 0.403; 95% CI = 0.205, 0.792). Respondents who do not have a current sexual partner were by 95% less likely to practice reproductive health rights than who have (AOR = 0.055; 95% CI = 0.025, 0.122), as displayed in Table 6.

## 4. Discussion

This study was intended to assess knowledge and practice of sexual and reproductive health rights and associated factors among Assela Preparatory School female students.

This study revealed that seventy percent of the respondents were knowledgeable about reproductive health rights. This finding is lower than the study conducted in Tanzania in 2013 [16]. The difference could be due to the study setting and health system linkage with educational sector differences. Media were the most reported source of information on reproductive health rights. A similar finding was reported in a study in Nigeria and Debremarkos [6, 13]. This indicates that media such as radio and televisions are the most common source of information about reproductive health in most developing countries which do not require any costing for accessing information compared to the Internet and newspapers. Especially, radio is accessible for most rural residents as it does not require electricity and works with a battery.

Ninety-seven percent of the respondents knew, at least, one sexually transmitted infection (STI) which was higher than the study conducted in Dire Dawa in 2014 which was 7.2% [17]. The participants from Dire Dawa were secondary school students, so there might low level of knowledge among those lower grade students compared to preparatory students. HIV/AIDS was reported by all of the respondents as the most known STI, followed by gonorrhoea. A similar finding was reported in a study conducted in Dire Dawa [17].

The majority (90%) of respondents mentioned contraceptive methods as a way of preventing unwanted pregnancy followed by abstinence (87.7%). Similarly, a study conducted in Dar es Salam showed that abstinence and condom use were reported as ways of preventing unwanted pregnancy [16]. Forty-six percent of the girls agreed on the families' right to decide on their female child marriage which was almost similar with the UNFPA report that most child marriages in Ethiopia are arranged by the parents [18]. More than two-thirds of the respondents oppose the idea of a married woman's right to refuse to have children if she does not want to. This indicates improvement of males' misperception toward males' dominance over female decision making power and current emphasis of family laws and other regulations toward females' equity in Ethiopia.

This study found that 94% of the respondents mentioned at least one modern contraceptive method, where injectable was the most known 91.2% method, and media 60.9% was a major source of information. This is in line with a study conducted in Dar es Salam where 97% of the respondents were knowledgeable about contraceptives. But, injectable was the most reported method in this study which was different from the study conducted in Dares Selam [16]. This might be due to the popularity of injectable contraceptive methods in Ethiopia. Sixty percent of girls discussed the reproductive issues with peers, which is similar to a study conducted in Dire Dawa [17]. But, from a study conducted in Nigeria, the majority of the study participants discuss with their parents [6]. This difference might be due to sociodemographic and cultural differences. Less than 25% of the respondents had used contraceptives, which was lower than the findings of Tanzania which was 40% [16]. The justification could be that awareness regarding sexual and reproductive issues by the schools might be well addressed Tanzania than our country. Injectable was the most utilized type, followed by emergency contraceptive methods. The reason could be that contraceptives such as injectables are taken once in an interval of three months which decreases daily memory, while pills are taken daily which is difficult to conceal from families or friends.

Unmarried respondents were 85% less likely to utilize modern contraceptives than the married (AOR = 0.155; 95% CI = 0.032, 0.762). A similar finding was reported from a previous study conducted in Ethiopia [15]. Mostly, married women use contraceptives to space births between deliveries or to limit the number of children they have as reproductive health rights. Most of the time, single girls do not engage in sexual intercourse; therefore, they do not worry about contraceptive use since they are not at risk of getting unwanted pregnancy.

Respondents whose fathers were farmers were more than 6 times more likely to utilize contraceptives; moreover, girls whose fathers were self-employed were more than 3 times more likely to utilize contraceptives than those employed by the government. The reason could be that these girls live away from the family in the rented house in town with limited control of the family. They may engage in sexual activity freely without fear of any family members; this may lead to the utilization of contraceptives. Those respondents who did not discuss on sexual and reproductive issues were 60% less likely to practice reproductive health rights (use contraceptives) than who discuss. During the discussion, they may be more familiar with different information on reproductive issues. Participants who do not have a current sexual partner during the survey were less likely to use contraceptives than their counterparts. This could be that girls without a partner most of the time do not engage to sexual activity and definitely they are not getting unwanted pregnancy and not use contraceptives. One of the limitations of this study was the absence of complementing with the qualitative method, which helps to explore a more sensitive issue. The other limitation could be the problem of a temporal relationship due to the design itself and social desirability bias.

## 5. Conclusions

In conclusion, in this study, majority of the respondents were knowledgeable about reproductive health rights and had a discussion on reproductive issues with their peers. The majority of the respondents knew, at least, one type of contraceptive method but with lower utilization. It is recommended that schools should establish clubs to promote sexual and reproductive health issues and rights. It also recommended working on awareness creation through multimass media and strengthening youth-friendly services in the area.

## Figures and Tables

**Table 1:** Sociodemographic characteristics of preparatory school female students, Assela, Ethiopia, in 2015.

Variable	Frequency	Percentage (%)
Age		
18	297	75.6
19	75	19.1
≥20	21	5.3
Mean		18.3 ± 0.57

Religion		
Orthodox	272	69.2
Muslim	77	19.6
Protestant	44	11.2

Residence		
Urban	238	60.6
Rural	155	39.4

Living with		
Mother or father only	67	17.0
Mother and father	170	43.3
Friends	29	7.4
Alone	61	15.5
Relatives	66	16.8

Marital status		
Single	380	96.7
Married	13	3.3

Father's education		
Illiterate	38	9.7
Elementary level	147	37.4
Secondary level	87	22.1
College/university	121	30.8

Mother's education		
Illiterate	59	15.0
Elementary	203	51.7
Secondary	70	17.8
College and above	61	15.5

Father's occupation		
Government-employed	138	35.1
Self-employed	72	18.3
Farmer	149	37.9
NGO	25	6.4
Other	9	2.3

Mother's occupation		
Government-employed	69	17.6
Self-employed	101	25.7
House wife	201	51.1
NGO	20	5.1
Other	2	0.5

**Table 2:** Knowledge of sexual and reproductive health right among preparatory school female students, Assela, Ethiopia, 2015.

Variables	Frequency	Percentage (%)
Know about RHR		
Yes	277	70.5
No	16	29.5

Source of information about RHR		
Media (TV/radio)	188	67.9
Peers	45	16.2
Health institute	44	15.9

Reason for lack of information		
Poor relation with friends	16	13.8
No discussion with parents	60	51.7
No mass media access	7	6.0
No clubs in the school	33	28.4

Know safe time of pregnancy		
Yes	226	57.5
No	167	42.5

Know problems of unsafe sex		
Yes	364	93.3
No	26	6.7

Types of problems of unsafe sex		
Unwanted pregnancy	340	92.1
STI	339	91.9
Abortion	248	67.2
Other	5	1.4

Right of mate selection without family consent		
Yes	333	84.7
No	60	15.3

Right to disagree on marriage arranged by families		
Yes	345	87.8
No	48	12.2

Right of a married woman to say no to have children		
Yes	124	31.6
No	269	68.4

**Table 3:** Knowledge of STI and contraceptives among preparatory school female students, Assela, Ethiopia, 2015.

Variables	Frequency	Percentage (%)
Know sexual transmitted infection		
Yes	382	97.2
No	11	2.8

Knowledge on types of STI		
Gonorrhea	314	82.2
Syphilis	293	76.7
Chancroid	178	46.6
HIV	382	97.2
Herpes simplex	96	25.1

Know ways of preventing unwanted pregnancy		
Yes	352	89.6
No	41	10.4

Methods of preventing pregnancy		
Abstinence	283	80.9
Contraceptives	307	87.7
Withdrawal	152	43.5

Have information about contraceptives		
Yes	373	94.9
No	20	5.1

Knowledge of types of contraceptives		
Pills	334	89.5
Injectables	340	91.2
Loop	333	89.3
Withdrawal	145	38.9
Other	5	1.3

Source of information about contraceptives		
TV/radio	227	60.9
News papers	36	9.7
Peers	66	17.7
Leaflet	44	11.7

**Table 4:** Sexual and reproductive health right practice among female preparatory school students, Assela, Ethiopia, 2015.

Variables	Frequency	Percentages (%)
Extent of violence against girls		
High	76	19.3
Less	167	42.5
None	150	38.2

Have ever had sexual intercourse		
Yes	208	52.9
No	185	47.1

Have a sexual partner currently		
Yes	215	54.7
No	178	45.3

History of pregnancy		
Yes	40	19.2
No	168	80.8

Is the pregnancy wanted?		
Yes	10	25.0
No	30	75.0

Discussion on sexual and reproductive issues		
Yes	217	55.2
No	176	44.8

Discussed with		
Peers	130	60
Family	47	21.6
Partner	40	18.4

Ever used contraceptives		
Yes	89	22.6
No	302	77.4

Type of contraceptive used		
Oral pills	24	27.0
Injectable	21	23.6
IUCD/loop	10	11.2
Emergency contraceptive	18	20.2
Condom	15	16.9

Source of contraceptives used		
Clinics	33	36.3
Pharmacy	26	28.6
Shops	5	5.5
Heath centers	27	29.7

**Table 5:** Factors affecting knowledge of reproductive health rights among preparatory school female students, Assela, Ethiopia, 2015.

Variable	Know RHR		Odds ratio (OR)	
	No (%)	Yes (%)	COR (95% CI)	AOR (95% CI)
Know safe time of pregnancy				
Yes	52 (23.0)	174 (77.0)	2.079 (1.340, 3.227)*∗∗*	**1.636 (1.006, 2.659)** *∗*
No	64 (38.3)	103 (61.7)	1	**1**

Know ways of preventing unwanted pregnancy				
Yes	89 (25.3)	263 (74.7)	5.699 (2.862, 11.349)*∗∗*	**3.277 (1.461, 7.351)** *∗∗*
No	27 (65.9)	14 (34.1)	1	**1**

Problem of unsafe sex				
Yes	100 (27.5)	264 (72.5)	3.600 (1.599, 8.103)*∗∗*	1.688 (0.641, 4.443)
No	15 (57.7)	11 (42.3)	1	1

Know information about contraceptives				
Yes	104 (27.9)	269 (72.1)	3.880 (1.542, 9.763)*∗∗*	2.26 (0.80, 6.35)
No	12 (60.0)	8 (40.0)	1	1

Discussion on sexual and reproductive issues				1.540 (0.953, 2.489)
Yes	51 (23.5)	166 (76.5)	1.906 (1.230, 2.955)*∗*	
No	65 (36.9)	111 (63.1)	1	

Have sexual partner currently				
Yes	45 (25.3)	133 (74.7)	1.457 (0.937, 2.266)	1.458 (0.896, 2.373)
No	71 (33.0)	144 (67.0)	1	1

Residence				
Rural	54 (34.8)	101 (65.2)	1	**1**
Urban	62 (26.1)	176 (73.9)	1.518 (0.978, 2.355)	1.533 (0.946, 2.484)

NB: COR = crude odds ratio, AOR = adjusted odds ratio; *∗p* value <0.05, *∗∗p* value <0.01.

**Table 6:** Factors affecting practice of reproductive health rights among preparatory school female students, Assela, Ethiopia, 2015.

Variable	Contraceptive use		Odds ratio (OR)	
	No (%)	Yes (%)	COR (95% CI)	AOR (95% CI)
Marital status				
Married	13 (30.8)	9 (69.2)	1	**1**
Single	300 (96.69)	80 (21.1)	0.119 (0.036, 0.395)	**0.155 (0.032, 0.762** *∗∗ * **)**
Residence				
Rural	111 (71.6)	44 (28.4)	1.700 (1.056, 2.738)*∗*	1.371 (0.589, 3.190)
Urban	193 (81.1)	45 (18.9)	1	1
Living with				
Father or mother only	57 (85.1)	10 (14.9)	1	1
Father and mother	135 (79.4)	35 (20.6)	1.478 (0.686, 3.185)	2.542 (0.936, 6.905)
Friends	19 (65.50	10 (34.5)	3.000 (1.083, 8.309)*∗*	1.261 (0.331, 4.793)
Lonely/alone	40 (65.6)	21 (34.4)	2.992 (1.273, 7.034)*∗*	0.947 (0.296, 3.031)
Relatives	53 (80.3)	13 (19.7)	1.398 (0.565, 3.457)	0.835 (0.254, 2.748)
Father's occupation				
Government-employed	124 (89.9)	14 (10.1)	1	**1**
NGO	16 (74)	9 (16)	2.797 (0.958, 8.165)	1.933 (0.459, 8.133)
Self-employed	56 (77.8)	16 (22.2)	2.531 (1.156, 5.540)	**3.618 (1.222, 10.716)** *∗*
Farmer	98 (65.8)	51 (34.2)	4.609 (2.411, 8.812)	**6.137 (1.945, 19.369)** *∗∗*
Other	10 (76.5)	8 (23.5)	2.531 (0.478, 13.387)*∗∗*	1.563 (0.133, 18353)
Mother's occupation				
Government-employed	61 (88.4)	8 (11.6)	1	**1**
NGO	72 (71.3)	29 (28.7)	0.131 (0.007, 2309)	1.897 (0.419, 8.592)
Self-employed	156 (77.60)	45 (22.4)	0.429 (0.23, 8.43)	1.685 (0.557, 5.102)
House wife	15 (68.2)	7 (31.8)	0.403 (0.024, 6.658)	1.302 (0.448, 3.781)
Father's education				
Illiterate	28 (73.7)	10 (26.30	1	1
Elementary	106 (72.1)	41 (27.9)	1.083 (0.483, 2.427)	1.450 (0.515, 4.088)
Secondary	65 (74.7)	22 (25.3)	0.948 (0.397, 2.260)	1.780 (0.552, 5.748)
College or above	105 (86.8)	16 (13.2)	0.427 (0.175, 1.042)	1.707 (0.431, 6.759)
Know RHR				
Yes	208 (75.1)	69 (24.9)	1.59 (0.92, 2.77)	0.608 (0.295, 1.252)
No	96 (82.8)	20 (17.2)	1	1
Discussion on sexual and reproductive issues				
Yes	150 (69.1)	67 (30.9)	1	**1**
No	154 (87.5)	22 (12.5)	0.054 (0.026, 0111)*∗∗*	**0.403 (0.205, 0.792)** *∗∗*
Have a sexual partner currently				
Yes	98 (55.1)	80 (44.9)	1	**1**
No	206 (95.8)	9 (4.2)	0.320 (0.188, 0.544)*∗∗*	**0.055 (0.025, 0.122)** *∗∗*

## Data Availability

The raw data stored in SPSS software and the full report of the document including the tools (questionnaire) can be obtained from the corresponding author on request.

## Abbreviations

AIDS: Acquired immune deficiency syndrome
AOR: Adjusted odds ratio
COR: Crude odds ratio
EDHs: Ethiopian demography survey
HIV: Human immunodeficiency virus
ICPD: International Conference on Population Development in Cairo
IUCD: Intrauterine device
MDGs: Millennium development goals
RHR: Reproductive health right
SPSS: Statistical package for social science
SRH: Sexual and reproductive health
STIs: Sexually transmitted infections
UNPFA: United Nations Population Fund Agency.
