# Corrigendum to “Reproductive Health Right Practice among Preparatory School Female Students of Assela Town, Arsi Zone, Oromia Regional State, Ethiopia”

**DOI:** 10.1155/2022/9864542

**Published:** 2022-07-04

**Authors:** Tigist Tafa, Mesfin Tafa Segni, Hailu Fekadu, Shimelis Adugna, Meselech Assegid

**Affiliations:** ^1^Department of Public Health, College of Health Sciences, Arsi University, Assela, Ethiopia; ^2^Schools of Public Health, Addis Ababa University, Addis Ababa, Ethiopia

In the article titled “Reproductive Health Right Practice among Preparatory School Female Students of Assela Town, Arsi Zone, Oromia Regional State, Ethiopia” [1], the order of the authors and the corresponding author have been corrected as above. Specifically, first and corresponding author has changed from Mesfin Tafa Segni to Tigist Tafa.
